# *In Vivo* Two-Photon Microscopy Reveals Sensory-Evoked Serotonin (5-HT) Release in Adult Mammalian Neocortex

**DOI:** 10.1021/acschemneuro.3c00725

**Published:** 2024-01-22

**Authors:** Gabriel Ocana-Santero, Adam M. Packer, Trevor Sharp, Simon J. B. Butt

**Affiliations:** † Department of Pharmacology, University of Oxford, Oxford OX1 3QT, U.K.; ‡ Department of Physiology, Anatomy & Genetics, University of Oxford, Oxford OX1 3PT, U.K.

**Keywords:** 5-HT, biosensor imaging, somatosensory cortex, two-photon microscopy

## Abstract

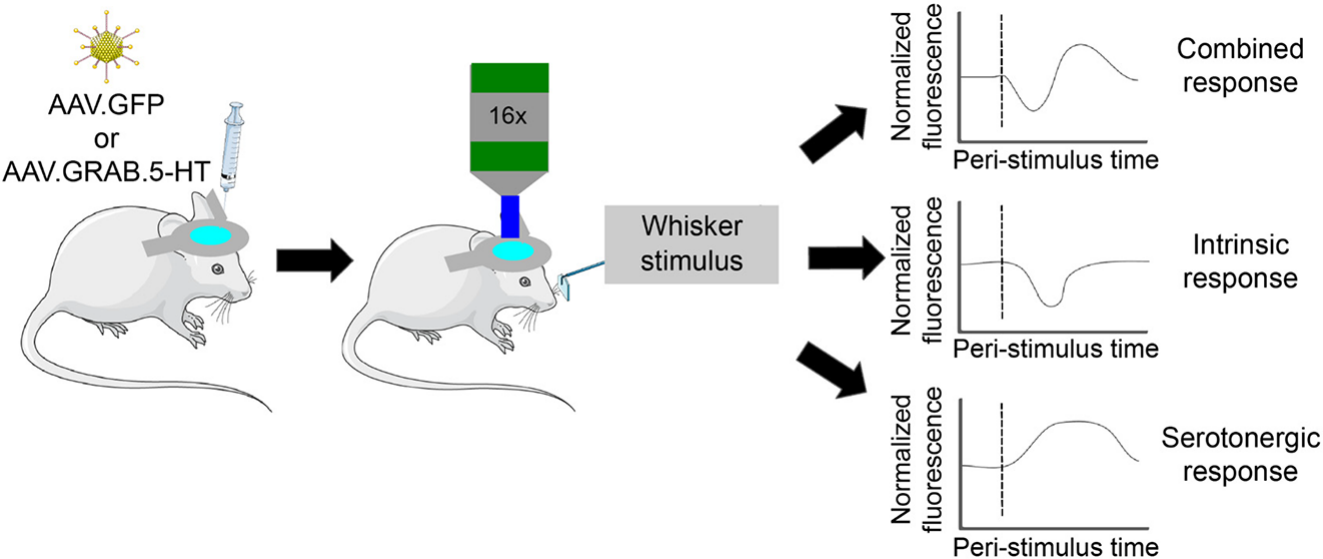

The recent development of genetically encoded fluorescent neurotransmitter biosensors has opened the door to recording serotonin (5-hydroxytryptamine, 5-HT) signaling dynamics with high temporal and spatial resolution *in vivo*. While this represents a significant step forward for serotonin research, the utility of available 5-HT biosensors remains to be fully established under diverse *in vivo* conditions. Here, we used two-photon microscopy in awake mice to examine the effectiveness of specific 5-HT biosensors for monitoring 5-HT dynamics in somatosensory cortex. Initial experiments found that whisker stimulation evoked a striking change in 5-HT biosensor signal. However, similar changes were observed in controls expressing green fluorescent protein, suggesting a potential hemodynamic artifact. Subsequent use of a second control fluorophore with emission peaks separated from the 5-HT biosensor revealed a reproducible, stimulus-locked increase in 5-HT signal. Our data highlight the promise of 5-HT biosensors for *in vivo* application, provided measurements are carried out with appropriate optical controls.

## Introduction

The recent development of neurotransmitter-sensitive fluorescent sensors generated from genes encoding receptors,^1−5^ when combined with multiphoton microscopy, has opened the door to monitoring 5-HT dynamics *in vivo* with high spatial and temporal resolution. In the past five years, a number of 5-HT fluorescent biosensors have been developed.^1−5^ These tools have been extensively validated *in vitro*, and studies to date suggest promise for monitoring 5-HT dynamics *in vivo*. Thus, changes in 5-HT signaling dynamics have been detected in cortical regions^1−3^ in response to alterations in the sleep–wake cycle^1,2,4^ and whole body movement.^3^ However, to date, few *in vivo* multiphoton imaging studies of 5-HT biosensors have controlled for the influence on the optical signals of confounding factors such as hemodynamic changes or other potential sources of optical noise which might limit selectivity of the signal and thus the sensitivity to the desired readout. While this source of noise is often considered and corrected in fiber photometry, it is less commonly accounted for in multiphoton imaging. Nevertheless, multiphoton imaging presents clear advantages over fiber photometry that merit its use in biosensor imaging. Namely, it provides a higher spatial resolution, with a readout of signal over space, including at depth within the tissue, thanks to its accurate optical sectioning. Cranial windows also represent a less invasive alternative to fiber implants. Altogether this warrants the testing and optimization of 5-HT biosensors in multiphoton imaging *in vivo*.

It is evident from previous calcium- and voltage-sensitive imaging studies that in settings where the changes of fluorescence are small (e.g., widefield imaging or use of sensors with a small dynamic range), activity-dependent optical signals can be a major source of noise.^6,7^ Such confounding intrinsic optical signals may arise from many sources including changes in blood flow (e.g., blood vessel dilation),^8,9^ differences in hemoglobin and oxygenation state,^9^ and alterations in local cellular activity,^6,10−14^ all of which alter the light absorption properties of the tissue imaged, in wavelengths overlapping with our emission spectra. Given their inherent activity-dependent nature,^15^ all these factors represent challenging confounds to overcome.

Interference from intrinsic optical signals has long been recognized as a potential issue when performing optical recordings of neural activity.^6^ A preferred control to account for this source of noise would be to use a second imaging laser tuned to the isosbestic point, the imaging wavelength at which the absorbance, and subsequent emission, of a sensor will not change independently of its conformation (i.e., bound or not to serotonin). Thus, changes in fluorescence at this wavelength can be attributed to noise rather than changes in 5-HT binding. Simultaneous imaging with one laser tuned to the isosbestic wavelength and another tuned to the peak in fluorophore excitation allows intrinsic artifacts to be subtracted, thereby revealing the true biosensor signal. This control is commonly implemented in fiber photometry^16^ but not often with multiphoton microscopy due to the technical complexity of aligning two raster-scanning systems in time and space.

Here, we used two-photon microscopy combined with GRAB-5-HT biosensors^1,4^ to test the applicability of specific 5-HT biosensors *in vivo* within the context of a relatively simple but still unresolved question: are sensory responses in mammalian neocortex accompanied by changes in 5-HT dynamics? Previous studies show changes in the firing of midbrain 5-HT neurons during the delivery of specific sensory stimuli,^17^ but the dynamics of 5-HT release in sensory cortical areas is unknown.

## Results and Discussion

**Figure 1:**
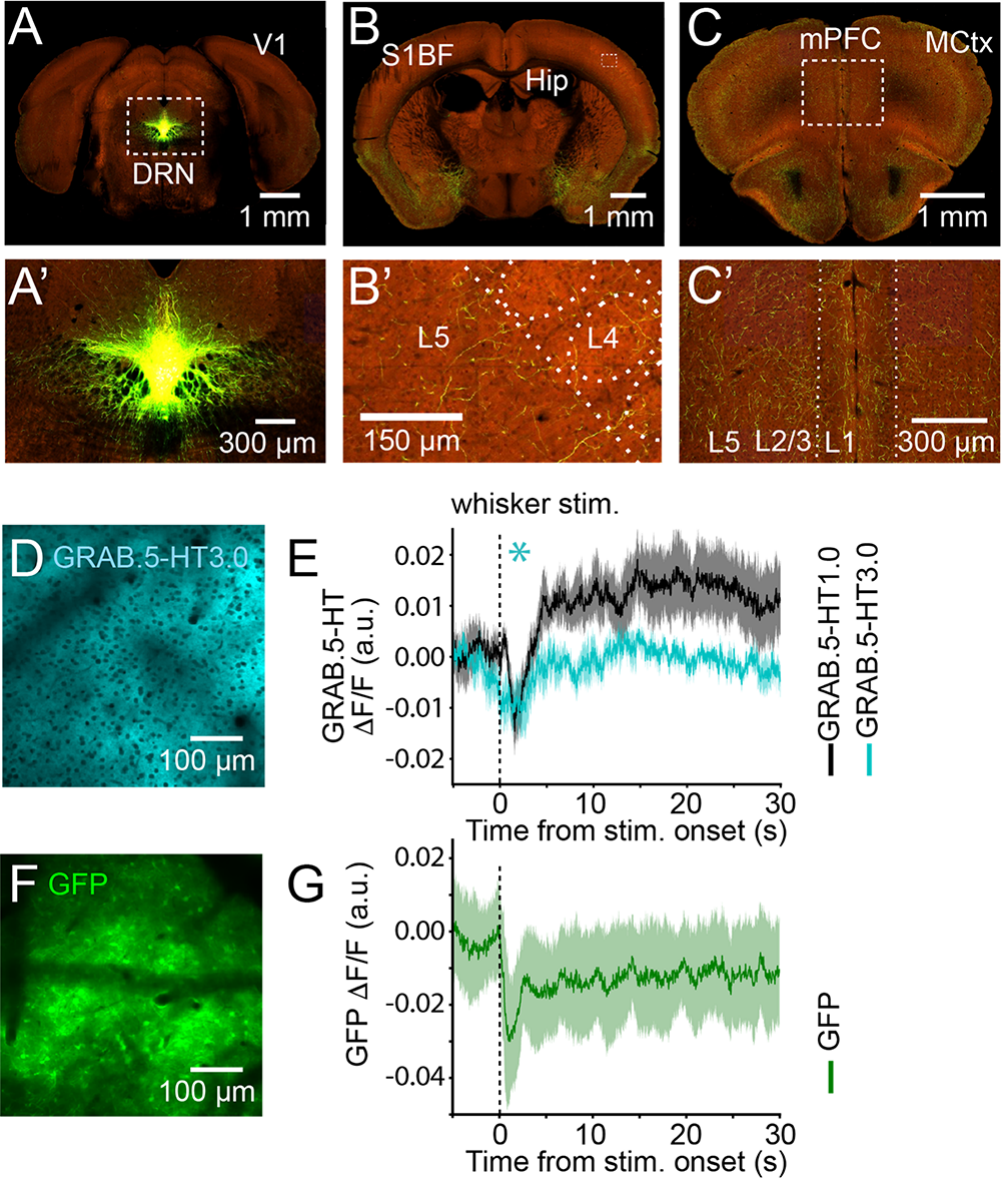
Effect of intrinsic optical signals on *in vivo* multiphoton imaging of 5-HT biosensor dynamics in the mouse somatosensory cortex. (A) Anterograde tracing of Cre-dependent EGFP generated by viral vector (AAV2/1.pCAG.FLEX.EGFP.WPRE.bGH) injection into the dorsal raphe nucleus of adult SERT-Cre mice showing the degree of 5-HT innervation in (B) somatosensory cortex (S1BF) and (C) medial prefrontal cortex (mPFC) (parts A′–C′, higher magnification images). All images used for parts A–C were obtained from the Allen Brain Projectomics Atlas.^19^ Expression of (D) GRAB.5-HT3.0 and (F) GFP in somatosensory cortex delivered using a viral vector under the human synapsin promoter. Peri-stimulus whole-field of view traces showing changes in (E) GRAB.5-HT1.0 (black trace, peak decrease at 1.2 ± 0.1 s poststimulus, *n* = 5) or GRAB.5-HT3.0 (cyan, peak decrease at 1.1 ± 0.4 s poststimulus, *n* = 3) and (G) GFP (green, peak decrease at 1.05 ± 0.2 s poststimulus, *n* = 3) in response to whisker stimulation (averaged across 10 stimulations); lines and shaded areas represent mean ± SEM values. “*n*” indicates the number of mice in each group. * (*p* < 0.05): mean signal 1 s poststimulus is significantly decreased compared to 1 s prestimulus (Shapiro test, statistic = 0.99, *p* = 0.82; paired *t* test, statistic = 5.82, *p* = 0.02).

Experiments tested the impact of whisker stimulation on primary somatosensory barrel cortex (S1BF) of adult mice, an established model of sensory encoding in a cortical region which is well-known to receive a 5-HT innervation albeit somewhat weaker than other cortical regions (Figure 1A–C). We injected into the S1BF viral vectors genetically encoding GRAB.5-HT1.0 or GRAB.5-HT3.0 two recently developed GPCR-based 5-HT biosensors engineered from 5-HT_2C_ and 5-HT_4_ receptors, respectively^4^ (Figure 1D,E). Mice were then imaged during periods of whisker stimulation using two-photon microscopy under awake, head-fixed conditions. Our experimental paradigm was well-suited to the study of possible contamination of the biosensor signal by intrinsic optical noise because whisker stimulation triggers strong neuronal activity accompanied by a robust intrinsic optical signal.^18^ As a control, other mice were injected with genetically encoded green fluorescent protein (GFP) which has no reported biosensor capability (Figure 1F,G).

It was found that whisker stimulation triggered a pronounced, short-latency decrease followed by a delayed but marked increase in the fluorescence signal in GRAB.5-HT-expressing S1BF (Figure 1E). However, the dynamics of this fast decrease in fluorescence (1.1 ± 0.1 s poststimulus; *n* = 8) were incompatible with the temporal resolution of the two 5-HT sensors tested (TauOFF GRAB.5-HT1.0 = 2.8 s;^1^ TauOFF GRAB.5-HT3.0 = 1.7 s ^4^). Indeed, repeating the experiment in animals injected with GFP expressing viral vector (Figure 1F) showed a similar decrease in fluorescence (peak decrease at 1.05 ± 0.2 s poststimulus, *n* = 3) after whisker stimulation (Figure 1G), suggesting that the fast drop in fluorescence is not related to changes in 5-HT dynamics. Similar sensory responses have been observed in primary visual cortex using widefield calcium imaging and have been associated with hemodynamic noise.^7^ Moreover, this finding aligns with prior literature indicating that reduced light reflectance links to increased neuronal activity.^6,10−14^

**Figure 2:**
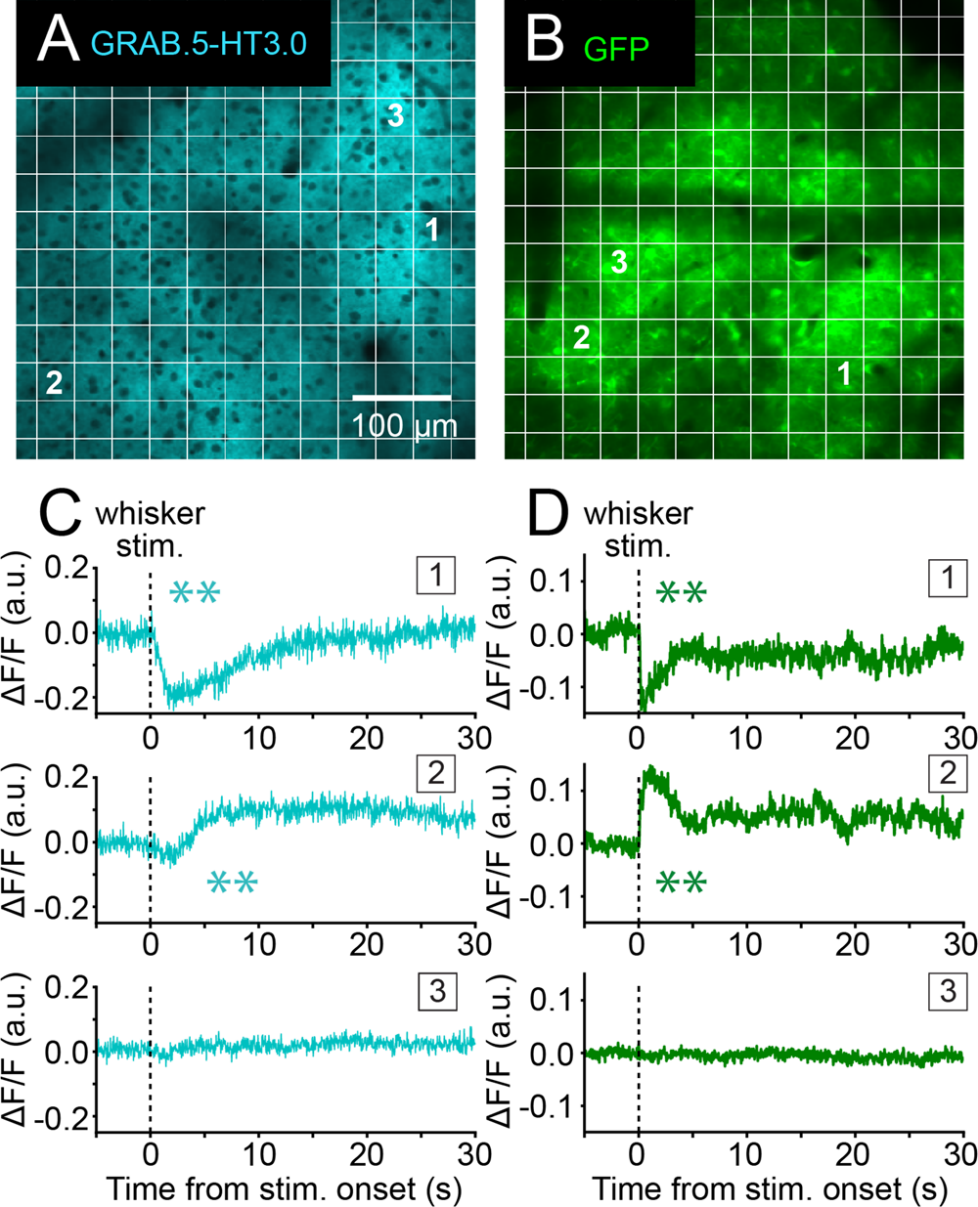
Whisker stimulus-evoked changes in intrinsic optical signals (GFP) and 5-HT biosensor signals show similar spatial variability. (A) GRAB.5-HT3.0 biosensor signal and (B) GFP fluorescence in field of view subregions of the S1BF. Examples of peri-stimulus traces showing a diversity of whisker stimulus-evoked changes in (C) GRAB.5-HT3.0 or (D) GFP in different subregions of S1BF from a single animal in each group imaged simultaneously. The numbers (1–3) match the traces to the corresponding subregions of the field of view. Same overall fields of view as shown in Figure 1. **Paired *t* test, *p* < 0.01 (GRAB.5-HT3.0, subregion 1, *p* = 0.002; subregion 2, *p* = 0.008//GFP, subregion 1, *p* = 6.20 × 10^–5^; subregion 2, *p* = 0.0001).

To explore the spatial complexity of the source of intrinsic optical noise within the somatosensory cortex, we quantified changes in fluorescence at a higher spatial resolution (S1BF field of view divided into 256 subregions; Figure 2A). This revealed variability in GRAB.5-HT3.0 responses to whisker stimulation across the field of view, indicative of the local changes in signal. Thus, a combination of transient increases and decreases in signal, as well as no change, was observed (Figure 2C). There appeared to be no meaningful spatial distribution to these signals across the imaging field of view with increases and decreases in the signal often observed in adjacent subregions. Similar response variability was observed in control GFP-expressing mice, with subregions that present increasing, decreasing, or unaltered fluorescent signals upon whisker stimulation (Figure 2B and D). This suggests that changes in the intrinsic optical signal might underlie the changes in the GRAB.5-HT biosensor signal. Thus, artifactual changes in the 5-HT biosensor signal suspected on the basis of their fast signal dynamics were confirmed by the observation of similar changes in 5-HT biosensor and GFP signals at high spatial resolution, which highlights the complexity of this optical source of noise.

**Figure 3:**
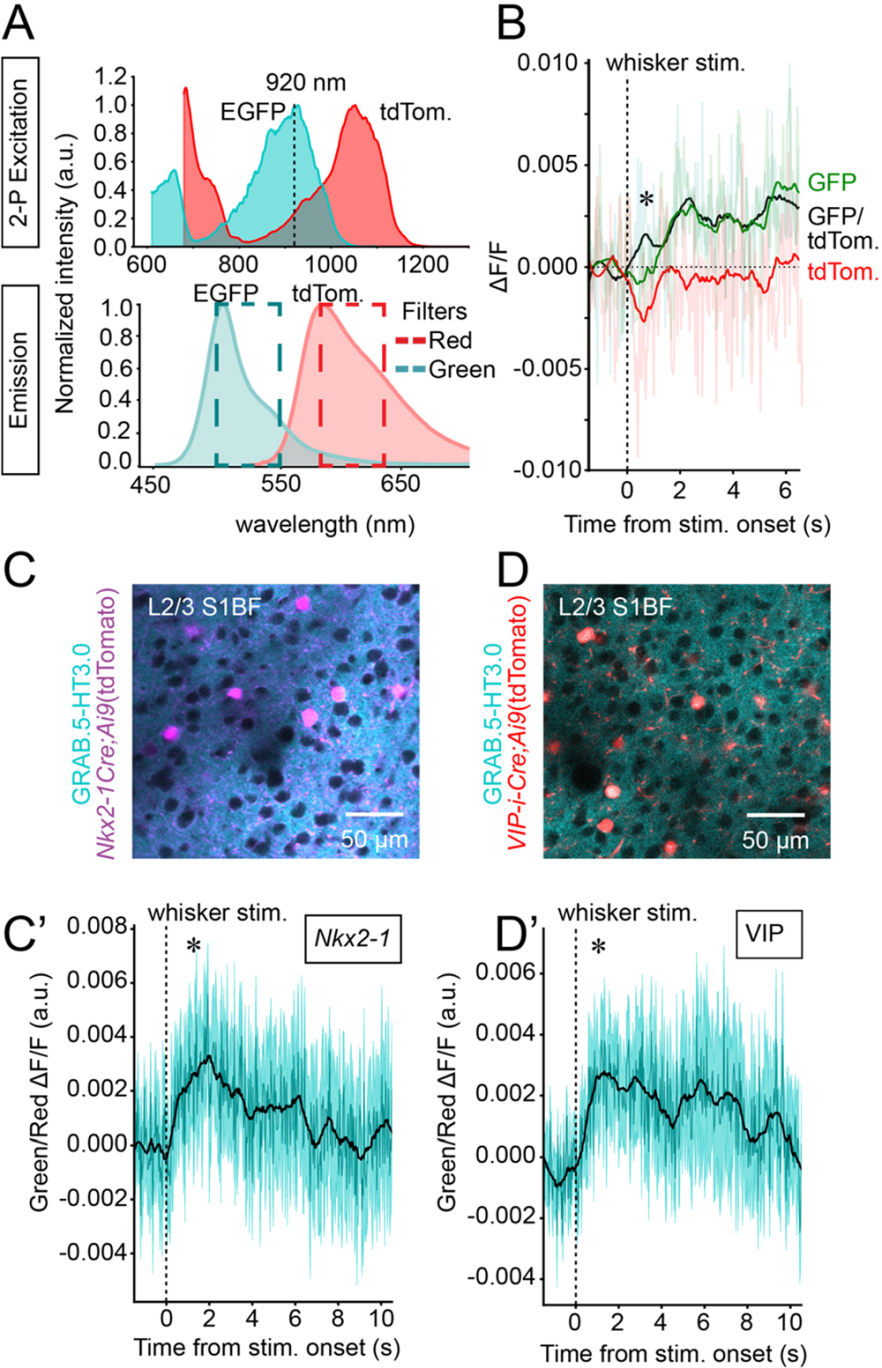
Evidence that dual-fluorophore imaging of GRAB.5-HT3.0 and a control red-fluorophore allows real-time hemodynamic correction when isosbestic correction is not possible. (A) Two-photon excitation (top) and emission (bottom) spectra of EGFP and tdTomato. A two-photon imaging wavelength of ∼920 nm stimulates both fluorophores, while their emission peaks are sufficiently separate to allow recording via different channels (e.g., green and red) without major cross-contamination. The cyan and red dashed line boxes indicate the cutoff wavelengths for the green and red emission filters, respectively (green, 525 ± 25 nm; red, 595 ± 25 nm), which allow separation of EGFP and tdTomato signals in different recording channels. Spectra plots made from data downloaded from the FPbase data set.^23^ (B) Peri-stimulus traces showing a whisker stimulus-evoked drop in red tdTomato signal, a smaller drop in green GRAB.5-HT3.0 signal, and an increase in the ratiometrically corrected signal (black). * (*p* < 0.05): mean signal 1 s poststimulus is significantly increased compared to 1 s prestimulus for the black trace (Shapiro test, statistic = 0.91, *p* = 0.28; paired *t* test, statistic = 3.03, *p* = 0.01). Traces are the average response to 10 stimuli presentations recorded from a single representative animal. (C) Field of view with GRAB.5-HT3.0 and tdTomato in *Nkx2-1* positive neurons (top) and (C′) peri-stimulus traces (bottom, *n* = 4). (D) Field of view with GRAB.5-HT3.0 and VIP positive neurons (top) and (D′) peri-stimulus traces (bottom, *n* = 3). Peri-stimulus traces are the average of 10 stimulus presentations for four *Nkx2-1*+ and three VIP+ animals. Shaded area represents the standard error of the mean, and the black trace represents the Savitzky–Golay filtered signal. “*n*” indicates number of mice in each group. * (*p* < 0.05): mean signal 1 s poststimulus is significantly increased compared to 1 s prestimulus (*Nkx2-1*, Shapiro test, statistic = 0.96, *p* = 0.80; paired *t* test, statistic = 3.23, *p* = 0.048//VIP, Shapiro test, statistic = 0.85, *p* = 0.24; paired *t* test, statistic = 4.64, *p* = 0.043).

To prevent the intrinsic optical signal obscuring changes in the 5-HT biosensor signal, we implemented a single-laser dual-fluorophore approach. Imaging of the 5-HT biosensor fluorophore simultaneously with a control fluorophore allowed subtraction of the intrinsic optical signal to reveal the 5-HT-specific signal. With a single laser the two fluorophores must have overlapping two-photon excitation spectra (Figure 3A, top) but distinguishable emission peaks that could be captured through separate recording channels (Figure 3A, bottom). Thus, we combined EGFP (the fluorescent protein of the 5-HT biosensor) and tdTomato, a red fluorophore that has previously been used to provide a readout of intrinsic optical signals^20^ (Figure 3A). To this end, we virally delivered GRAB.5-HT3.0 to the S1BF of transgenic mice expressing tdTomato in either vasointestinal peptide (VIP) or *Nkx2-1* positive GABAergic interneurons, the latter accounting for approximately 10% of the neurons in the field of view (Figure 3C,D). Indeed, both transgenics allowed targeting of populations present within our field of view, partially overlapping with the expression of the GRAB biosensor (under the control of the pan-neuronal hsyn promoter) and with widespread neuropil labeling thanks to the dense innervation exerted by these interneurons, which makes them ideal transgenics for the targeting of our control fluorophore.

Since we were expecting to record relatively small changes in fluorescence^4^ (Figure 1B,B′), we utilized a smaller field of view, which at equal frame rate increased the dwell time per micron of tissue, thus increasing the signal-to-noise ratio. The dual-color recordings revealed a whisker-evoked drop in signal in the red fluorophore (Figure 3B) as previously observed with a GFP signal (Figure 1G), confirming its utility as a control. By calculating the ratio of the green to red signal, it was possible to correct for the putative hemodynamic response and reveal a whisker-evoked increase in GRAB.5-HT3.0 signal (Figure 3C′,D′). These results are consistent with a human PET study showing increases in occipital cortex 5-HT release upon the presentation of visual stimuli^21^ and suggest that 5-HT may be involved in sensory processing.^22^ We measured comparable 5-HT responses in both VIP and *Nkx2-1* transgenic mice (Figure 3C′,D′), suggesting that both serve as an appropriate control.

In conclusion, we recorded an increase in 5-HT biosensor dynamics in mouse somatosensory cortex in response to whisker stimulation using an approach that allows segregation of 5-HT signaling dynamics from confounding intrinsic optical signals. This was achieved using expression of a control fluorophore (tdTomato) in a subset of cortical neurons, which allows us to account for this source of noise across the field of view. These results validate the use of fluorescent biosensors to measure serotonin dynamics *in vivo* with multiphoton imaging, when using appropriate controls. We hope this work will open the door to future endeavors studying serotonin dynamics with unprecedented spatial resolution, at both the population and cellular level. Finally, we advocate the use of control fluorophores such as those applied here to account for intrinsic optical signals in future *in vivo* two-photon 5-HT biosensor studies and two-photon biosensor studies more generally.

## Methods

### Animals

Experiments were approved by a local ethical review committee at the University of Oxford and covered by UK Home Office licenses PE5B24716 and PP8136190. Mice were housed in a temperature-controlled room under a 12 h light/12 h dark cycle with free access to food and water ad libitum. Male and female adult C57Bl6 mice between 8 and 12 weeks were used throughout. Mice expressing tdTomato in select neuronal populations were generated by crossing Cre-driver line males, either *Nkx2-1-Cre* (*Tg(Nkx2-1-cre)1Wdr*) (MGI:3761164) or *VIP-Cre* (*Vip<tm1(cre)Zjh>*) (MGI:4431361), with females homozygous for the *Ai9* tdTomato reporter allele (*B6.Cg-Gt(ROSA)26Sor tm9(CAG-tdTomato)Hze/J*) (MGI:3809523).

### Viral Vector Delivery

Intracerebral delivery of viral vectors was performed as previously described.^24^ In brief, mice were anaesthetised with isoflurane, head-fixed in a stereotaxic frame and a 1 cm incision was performed on the scalp prior to craniotomy (coordinates AP = −1.9, ML = 3.0 from bregma). Viral vector solutions were loaded into a glass micropipet, and 300 nL was injected over 3 min at 300 μm from the surface of the brain. After a further 3 min, the micropipet was withdrawn, and the wound was closed with absorbable surgical stitches. Mice were then placed on a heating pad until recovery. Data included in Figure 3 came from adult animals in which the viral vector was injected at neonatal ages (coordinates AP = 1.4, ML = 1.5 from lambda and DV = 0.3–0.6 from the surface of the skull) to enable longitudinal tracking of GRAB sensory expression.

Viral vectors were as follows: AAV9.hsyn.GRAB.5HT3.0 (WZBiosciences, titer ≥1 × 10^13^ vg/mL), pAAV.hsyn.GRAB.5-HT1.0 (Addgene 140552-AAV9, titer ≥1 × 10^1^^3^ vg/mL), and pAAV.CAG.GFP (Addgene 37825-AAV1, titer ≥7 × 10^1^^2^ vg/mL).

### Cranial Windows

1–2 weeks after injection, cranial windows were generated as described previously.^25^ In brief, mice were anesthetised with isoflurane, head-fixed in a stereotaxic frame and the scalp was removed to allow placement of a head-fixing plate which was fixed to the skull using dental cement (Superbond, Sun Medical). After craniotomy (3 mm diameter) over the S1BF (center of the window AP = −1.9, ML = 3.0) two coverslips (3 and 4 mm diameter) were attached to each other with optical glue and placed over the craniotomy, sealed with vetbond, and immobilized with dental cement. Mice were then placed on a heating pad until recovery. For neonate animals (see above), a similar procedure was performed during the second postnatal week (window coordinates AP = 2.4 and ML = 2.8).

### Two-Photon Imaging

Animals were imaged at least 4 weeks after vector injection and window implantation and up to 10 weeks postinjection in the case of neonatal injections. Good expression was confirmed before imaging based on visual inspection and raw average signal in the green channel. Two-photon imaging was performed on a resonant galvo scanning two-photon microscope (Bruker) with a Chameleon Ultra II laser (Coherent) and 50 mW of power on sample. A 16×/0.8 NA water immersion objective lens (Nikon) was used. GRAB.5HT3.0, GRAB.5HT1.0, GFP, and tdTomato were imaged by using a 920 nm beam. Imaging was performed at 30 Hz in a squared field of view (Figures 1 and 2, 643 μm × 643 μm, or Figure 3, 287 μm × 287 μm). All recordings were obtained ∼150 μm from the brain surface (cortical layer 2/3). Sensory stimuli were generated pseudorandomly using Matlab customized code. Whisker stimulation was delivered with a piezoelectric actuator connected to a custom-designed (3D-printed) whisker stimulator. All traces were time-locked using PackIO^26^ (lab custom-designed software). All mice were imaged during adulthood, at least 3-weeks after viral delivery to ensure high expression of the GRAB biosensors and GFP.

### Analysis

All imaging data were preprocessed by registration with Turboreg^27^ against a mean-intensity average of 200 frames in ImageJ (Fiji).^28^ All fluorescent (*F*) traces are presented as Δ*F*/*F*:
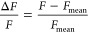
where *F*_mean_ is the mean fluorescence across the length of the recording. For dual-color experiments (Figure 3) the signal from tdTomato was used as a ratiometric control:
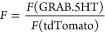
Signal filtering was performed using the Savitzky–Golay filter with a third order polynomial on windows of 31 frames (∼1s) for averaged traces (i.e., Figure 3C′,D′) and 61 frames (∼2s) for single animal traces (i.e., Figure 3B).

### Statistics

Statistical significance in the responsiveness was assessed by comparing the mean signal on a prestimulus window of time (−1.5 to −0.5 s prestimulus) against the mean signal on a poststimulus window (0.5 to 1.5 s poststimulus) across 10 stimuli. Comparisons were made using the Wilcoxon signed-rank test or the paired samples *t* test, after assessing non-/normality with the Shapiro–Wilk test.

### Software

Software microscopy images were processed with Fiji (processing package based on ImageJ).^28^ Figures were mounted and labeled using Inkscape. Images from the graphical table of contents were obtained from SERVIER medical arts kits (https://smart.servier.com/). All analysis, statistics, and plotting were performed in python 3.9.7.
